# Lectures on Typhoid Fever, Delivered in the Hotel Dieu Hospital of Lyons

**Published:** 1849-06

**Authors:** 


ART. II.—*Lectures on Typhoid Fever, delivered in the Hotel Dieu Hospital of Lyons.* By M. BRACHET. Translated from the French, by DAVID W. YANDELL, M.D., of Louisville, Kentucky. (Continued.)

The disease presents, as you will have remarked, three distinct and well marked periods or stages. The first is characterized by a more or less intense cephalalgia, insurmountable lassitude, sad and sunken countenance, loss of appetite, tongue white and sweetish, abdomen slightly painful, occasionally diarrhea, pulse quick and sharp, gums coated with a pearly white.

The second period is but an aggravation of the first: the cephalalgia becomes more obtuse, or even entirely disappears, or rather the patient no longer takes cognizance of it. Coma-vigil comes on; the intellectual and moral faculties grow more and more vitiated and deadened, and revery and delirium supervene and continue to augment. The expression of stupor and heaviness reaches the highest degree; the prostration is extreme; the patient is no longer able to remain in any other position than upon his back; copious sweats supervene and develop sudamina; the tongue becomes brown and covered, in common with the teeth and lips, with a fuliginous coat; finally, the skin of the patient is observed to be more or less studded with a rosy lenticular eruption; the diarrhea generally augments, though it is sometimes suspended; the dejections are also often involuntary.

As the second period is but an aggravation of the symptoms of the first, the third period is but an aggravation of the second, the symptoms in this gradually attaining their maximum of intensity; the moral and intellectual faculties are entirely abolished; all the senses become more obtuse; some are even apparently destroyed. To the coma-vigil or typhomania, sometimes succeeds somnolency, and sometimes coma and carus. The power of motion is so feeble that the patient remains almost immovable in bed, to the foot of which he seems to glide by his own weight; deglutition becomes difficult; the alvine and urinary discharges are involuntary or suspended; the mouth is filled with fuliginous matter; the heart beats with an almost incalculable rapidity; a viscid sweat covers the skin; intestinal hemorrhages sometimes arise; and it is at this period also that ecchymoses occur, and eschars are developed upon the trochanters, sacrum, feet, and genital organs.

From what I have already said of the progress and signs of typhoid fever, and from the cases that you have had an opportunity of seeing daily, you are, I trust prepared to admit that it is an essential and distinct disease; and, what is equally if not more important, able to distinguish it from the other affections with which it has often been confounded.

Although this would be the proper place to speak of the terminations of typhoid fever, I believe it preferable first to develop my opinions of its character or essence, since this study will serve to throw much light upon, if not to explain, many of the peculiarities of its terminations, whieh have not yet been correctly understood. What, then, is the character, or, in other language, the nature of typhoid fever? The solution of this important question requires, first, that we establish the seat of the disease, or that we determine the organ or organs principally affected. There are two ways of doing this—by cadaveric inspection, or pathological anatomy; and by the examination of functional lesions or pathological physiology.

What, now, does the dead body teach us? What lesions do the different organs of the body present? In the two bodies that have been examined in your presence you have had a complete anatomical table of the disease,

In the cranium we found a slight serous infiltration within membranes, and especially in the pia mater, which appeared in some points somewhat congested. The arachnoid presented nothing abnormal, though in some cases it is more opaque than in health, and offers traces of phlogosis. The vessels appeared slightly injected, though not more so, or, indeed, quite so much, as in many other diseases, unaccompanied by any cerebral lesions whatever. The substance of the brain is entirely healthy, save, probably, a slight increase in its consistency. In no portion of its structure have we met with either red or diffluent softening, suppuration or effusion; in slicing it, however, it has always appeared speckled with small drops of blood.

These, the ordinary physical lesions of this organ in typhoid fever, are hardly ever met with in the brain of persons who have died of disease in which the brain has borne no part. We have never found them in the same degree, except in cases of rheumatismal metastasis upon the encephalon. Certainly these fall very far short of explaining the cerebral phenomena of the disease, and still less the death which so often proceeds from the brain when it takes place towards the end of the first week or in the course of the second week.

In one instance only have we examined the medulla oblongata. The slight injection and infiltration which we found, and which have been observed by others, throw no additional light upon the seat of the disease. It cannot be located there.

The inspection of the nerves has never revealed anything to us abnormal. On the part of ganglionic life, the heart has offered nothing noticeable. We are unable to confirm the observation of certain authors who profess to have found a species of softening in the tissue of this organ. The appearance of the arteries and veins has been alike always normal. So much cannot be said of the capillaries. This portion of the circulating apparatus has appeared to us always more or less congested, especially in the first two, and sometimes in the third week. This explains why, at this period, the conjunctivas, lips, gums, tongue, palate, and entire length of the mucous membranes, are injected. This is the reason why the parenchymatous organs, which are largely supplied with capillaries, such as the lungs, spleen, and even the liver, are generally congested and someties softened. It is also owing to this cause that the cellular tissue preserves its fullness and roundness during this period of the disease. And it is also, perhaps, to this condition of the capillaries that we may, in part at least, attribute the hemorrhages, and especially the cutaneous and intestinal eruptions of which we have spoken. It is necessary to observe, however, that too much importance must not be attached to this phenomenon; for there is in these eruptions something more than a pure and simple effect of capillarity; there is something special which can be explained only by a vital modification altogether peculiar. This state of capillarity alone does not constitute the disease. Death is rarely the consequence of it; this occurs only in cases which we shall presently indicate to you.

We have already described the rose colored, lenticular spots of the integuments. It is necessary to remark, however, that although this eruption is peculiar, and, when it exists, is pathognomonic, it does not fix the disease in the integuments, much less constitute it, since it is never present from the beginning, and is sometimes entirely absent during the whole progress of the disease. Further, of what importance, what part can these rose colored patches perform in the production of typhoid phenomena?

The morbid condition of the intestinal mucous membrane, and especially of the inferior portion of the ileum, is of much greater importance. The intestine of No. 13, which is before you, presents a union of all the various lesions that have been pointed out; the figured patches, grey and hard; the reticulated and soft patches; the congregated pustules, the scattered follicles, the black spotted patches; and, in many points, ulcerations, some formed, others commencing; while portions of the figured patches appear to be affected with gangrene, and form strips or flaps still adherent, but floating and ready to be detached. Is this gangrene, as the greater number of authors have asserted? No; it in no wise resembles it. This pustular eruption, analogous to the pustular eruptions of the skin, presents in its structure a diseased portion, which, in most cases, becomes detached and falls off. If it were not in a situation moistened by liquids, it, as the eruption on the skin, would dry up; but the mucus which continually bathes it softens, and, in the end, detaches it. Let not the perforation of the intestine be urged in proof of the gangrene. It is sufficient to overthrow this, to refer you to what is going on at this moment in many of our smallpox patients.

We have repeatedly called your attention to every variety of termination of pustules. Some, while still but slightly developed, dry up and disappear in some degree by resolution, without appreciable detritus. The greater number, however, form a thick crust, which, at its fall, reveals the participation of the dermis in this pitted cicatrix, which proceeds from a loss of substance. Among some of the patients, you have seen the ulcerated dermis traversed throughout its entire thickness by the cicatrix; while, in a smaller number, the pustule involves in its formation the sub-cutaneous cellular tissue, and at its fall leaves an ulceration of considerable depth.

Now what you have seen in smallpox, occurs in like manner in the typhoid eruption of the intestines. This eruption, as a general rule, is constant, though it is not always so extensive nor so varied as you now see it.

There are often but two or three figured patches, or a few scattered pustules, so that this form of intestinal lesion may be regarded as pathognomonic, in as much as since the time of Rœderer and Wagler, who were the first to distingush it, and especially since MM. Petit and Serres, who gave it the name of entero-mesenteric fever, down to the learned researches of MM. Bretonneau, Louis, Chomel, Forget, etc., it has been made, so to speak, the battle field of typhoid fever, many authors having located there the entire disease; some under the name of dothinenteritis, while others called it follicular and capillary enteritis. Although perforations of the intestine occur, as we have said, most frequently at the onset, they are sometimes affected through an ulceration which at first, occupies but a portion of the thickness of the intestine, and which gradually corrodes it, whilst it sometimes happens that the base of the ulceration is suddenly destroyed by a strain or the passage of some intestinal matter.

It would be erroneous to deduce from what we have said, that it is impossible for the disease to terminate by gangrene of the intestine. We know that gangrene sometimes supervenes from the intensity, and oftener, from the malignity of the inflammation, but we have desired to call your attention only to what most generally occurs.

However much disposed we may be to regard this lesion of the intestine as an apanage of typhoid fever, we nevertheless do not believe it correct to place the entire disease in this eruption. No; typhoid fever is not, and cannot be, a simple dothinenteritis, a follicular enteritis.

In the cases most commonly met with, the digestive tube is not diseased to the extent of producing death, nor even the pathological phenomena of the disease. Furthermore, this eruption never exists at the commencement of the disease. A greater or less length of time always elapses before it makes its appearance. How, then, under these circumstances, can this local lesion be the cause of the other accidents? Would this not be to admit an effect without, or at least before, a cause? If we found the opinion upon the fact that, at a later period, these patches are the cause of ulcerations which produce death, we attribute to them a part which is not properly theirs, as we shall presently see.

Let us pass now to the pathological condition of the mesenteric glands, which are found in this disease of every known condition, varying in size from that of a pea to that of a nut; in color, from an inflammatory rose, to the dark red bordering upon gangrene; from the first traces of purulent infiltration to the almost total dissolution of the tissues of certain of them. Their lesion, however, is neither so constant nor so important as that of other tissues or organs; it is always secondary, and is always the effect of the disease of the intestine, unless they (the mesenteric glands) were previously diseased, because of tubercles, scrofula, or some other lesion. However valuable these anatomical data may be, especially those relative to the encephalon, capillaries, and follicular eruption of the intestine, they are insufficient to explain the disease, to give the key, to enable us to locate it exclusively in a single organ, and are still less competent to reveal to us its nature. Let us see, now, if we shall be any more fortunate in the province of physiological pathology, and if, in associating it with anatomical lesions, we shall be able to arrive at a satisfactory solution of the question. You are aware of our great confidence in pathological physiology. This language of the diseased economy, this cry of the suffering organs, never deceives us. It is an *effect*, and in proceeding from the effect to its cause, we arrive by the analysis of the fact, or of the morbid act, at the cause, at the organ, and the lesion of this organ. Now, then, what have we observed in typhoid fever?

On the one hand we have cerebral phenomena of the highest interest, since they can proceed but from an organ profoundly diseased; since they are capable of producing and often are the cause of death. It may be asked if the encephalon does not execute these acts by a sympathetic reaction received from other diseased organs since it presents itself such insignificant physical lesions? If the cerebral phenomena were but the effects of a reaction upon the brain, they would be neither so uniform nor so unequivocal as they are; they would be varied, as are all the phenomena of sympathy or of reaction of any organ whatever upon the brain. Furthermore, this viscus would not be so seriously compromised as it is, and especially would it not be so frequent a cause of death. It is only in exceptional cases that this organ is so dangerously affected when merely the effect of sympathy. Hence there must be something besides the effect of reaction, something more than sympathy; it must itself be diseased. Such is the logical and necessary conclusion at which we arrive by this physiological reasoning. It can no longer be doubted that the brain is diseased, and it is because of this that it performs the vitiated acts which depend upon it. But is it alone diseased? Does it alone constitute the disease? This is what we now proceed to inquire.

We know that the capillary system is extensively involved, and we have attributed to this a number of phenomena, both on the part of the skin, mucous membranes, and substance of certain parenchymatous organs. Universally disseminated, it may operate universally in carrying its action and its influence into all the tissues. And yet, without denying the active and considerable part that it bears in typhoid fever, we are very far from esteeming it the seat or sole agent of the disease. It is not this which produces the cerebral accidents, for, in many instances, the capillarity of the brain is found augmented without the existence of any typhoid symptoms. Nor are the variations of the circulation to be attributed with any more reason to this cause, from the fact that it frequently happens that much greater capillary congestions exist without the heart presenting these anomalies. Finally, it cannot be this which occasions dothinenteritis, since it is often the case that the intestine is the seat of more extensive congestions, which do not produce it when the typhoid poison is not the cause. This condition of the capillaries, however, merits our most careful attention, because of the important part it plays; but it constitutes neither the exclusive seat of the disease nor the agent of its phenomena.

